# Sexually transmitted infections in women with fertility problems in Aragua, venezuela

**DOI:** 10.17843/rpmesp.2022.392.10889

**Published:** 2022-06-20

**Authors:** César Pacheco, Jham Lugo, Narviz Pulido, Heriberto Correia, Nancy Moreno, Flor Herrera

**Affiliations:** 1 Instituto de Investigaciones Biomédicas (BIOMED) «Dr. Francisco J. Triana Alonso», Facultad de Ciencias de la Salud, Núcleo Aragua, Universidad de Carabobo, Venezuela. Universidad de Carabobo Instituto de Investigaciones Biomédicas (BIOMED) «Dr. Francisco J. Triana Alonso» Facultad de Ciencias de la Salud, Núcleo Aragua Universidad de Carabobo Venezuela; 2 Centro de Fertilidad CenFer, Clínica Lugo, Maracay, Estado Aragua, Venezuela. Centro de Fertilidad CenFer Clínica Lugo Maracay Estado Aragua Venezuela

To the editor. Some sexually transmitted infections (STIs) can cause infertility in women ^1^. In Venezuela there are few published reports on the proportion of these infections in women with fertility problems; therefore, there is a need to increase knowledge on this subject. The aim of this study was to determine the proportion of STIs due to *C. trachomatis, T. vaginalis, N. gonorrhoeae, *and* Mycoplasmas *(*M. hominis, M.genitalium *and* U. urealyticum*) in a group of women with fertility problems who attended the Fertility Center (CenFer) located in the city of Maracay, Aragua state during the years 2016-2019.

We conducted a cross-sectional study, with a continuous selection sample and non-probabilistic sampling. Infertility was defined according to the World Health Organization (WHO) ^2^. Heterosexual women who were sexually active, of reproductive age with primary or secondary infertility, nulliparous, with previous children or with history of previous or anembryonic abortions were included. Women on antibiotic treatment seven days prior to sampling were excluded.

The cervical swab sample was transferred from CenFer to the Biomedical Research Institute (BIOMED) for molecular diagnosis. DNA from the swabs was extracted with the phenol-chloroform method, its integrity was determined by electrophoresis in 1% agarose gels and its concentration by absorbance at 260 nm. DNA amplification was carried out using the method described by the manufacturer of the commercial Seeplex® STD6 ACE detection kit (Seegene, Seoul, Korea). This kit contains six specific primer pairs to detect single genes of the pathogens *C. trachomatis, T. vaginalis, N. gonorrhoeae, M. hominis, M. genitalium* and *U. urealyticum*; positive DNA controls are included. Amplification products were visualized by electrophoresis on 2% agarose gels. The sample was considered positive if it had one pathogen, negative if it had none, and mixed if it had two or more pathogens.

Absolute and relative frequency distributions were calculated for the results of the species identification tests and the chi-square (χ2) homogeneity test was used to compare the proportion of each of the infections between the studied years. Data were processed with RStudio 1.1.463 statistical software.

The average age was 33.6 ± 7.8 years with a range of 18 to 50 years. Table 1 shows the percentages of positive, mixed and negative samples, and the frequency distribution of pathogens in the most mixed positive samples, during the 2016-2019 period. The χ2 homogeneity test showed that the annual proportions of positive and negative samples, as well as the species identified, were not homogeneous for the studied period (χ2=35.96; p<0.001 and χ2= 33.36; p=0.003, respectively).

**Table 1:** Proportion and distribution of pathogens detected in women with fertility problems during 2016-2019.

	2016 n=125 (%)	2017 n=144 (%)	2018 n=101 (%)	2019 n=123 (%)	Total N=493 (%)	p-value a
Sample						
Positive	80 (64.0)	86 (59.7)	35 (34.7)	55 (44.7)	256 (51.9)	
Mixed	12 (9.6)	20 (13.9)	9 (8.9)	8 (6.5)	49 (9.9)	<0.001
Negative	33 (26.4)	38 (26.4)	57 (56.4)	60 (48.8)	188 (38.2)	
Pathogens in the most mixed positive samples						
*M. hominis*	29 (23.2)	32 (22.2)	19 (18.9)	33 (26.8)	113 (22.9)	0.034
*U. urealyticum*	32 (25.6)	39 (27.1)	10 (9.9)	5 (4.1)	86 (17.4)	0.001
*C. trachomatis*	18 (14.4)	15 (10.4)	6 (5.9)	16 (13.0)	55 (11.2)	0.392
*N. gonorrhoae*	6 (4.8)	9 (6.3)	1 (0.9)	3 (2.4)	19 (3.9)	0.676
*T. vaginalis*	4 (3.2)	7 (4.9)	3 (2.9)	5 (4.1)	19 (3.9)	0.922
*M. genitalium*	3 (2.4)	4 (2.7)	5 (4.9)	1 (0.8)	13 (2.6)	0.138

a Chi-square test for homogeneity.

The percentage of positive samples was high in 2016 (64.0%) and 2017 (59.7%) and decreased in 2018 (34.7%) and 2019 (44.7%). There was a shortage of health supplies during 2016-2017, and during 2018-2019 the import of these supplies increased ^3^. Quite possibly, our results were influenced by sociopolitical-economic factors that occurred in Venezuela during the 2016-2019 period ^3^. Retrospective studies are needed to corroborate these findings.

The percentage of C. trachomatis was 11.2%, similar to that found in a study performed in infertile women (11.5%) (4), which suggests a participation of this pathogen in the infertility of some patients. 

The percentages of *N. gonorrhoeae, M. genitallum*, and *T. vaginalis* were lower than those found in other studies on infertile women. Literature reports conflicting results for *U. urealyticum* and *M. hominis*. A study on infertile women found that the percentage of *U. urealyticum* and *M. hominis* was 51.7% and 26.7%, respectively ^5^. Another study in infertile and fertile women reported low percentages: *U. urealyticum* (6.6% infertile and 2.5% fertile) and *M. hominis* (5.3% infertile and 1.5% fertile) ^6^. Our study found percentages of 17.4% *for U. urealyticum* and 22.9% for *M. hominis*, these values more closely resembling the group of infertile women. In addition, these last two pathogens were the only ones that had a statistically significant annual variation, *U. urealyticum* decreased over time while *M. hominis* increased. It would be important to determine the cause of this behavior.

A limitation of our study is that the sample consisted only of women with fertility problems, so the data cannot be generalized to the entire population of Venezuelan women.

Finally, positive samples for STIs were higher in the first two years of the 2016-2019 period. This could have multiple causes such as a major shortage of drugs due to the sociopolitical-economic crisis in the country ^3^, the increase in the number of sexual partners or the lack of interest in timely and preventive urogynecological consultation. Of all these possibilities, it can only be confirmed that during the years 2016-2017 there was an 80% shortage of health supplies ^3^ and this could have influenced the increase in STIs.
