# Supplementary material for: Sexually transmitted infections in women with fertility problems in Aragua, venezuela
Source: Rev Peru Med Exp Salud Publica. 2022 Jun 20;39(2):247–8. doi: 10.17843/rpmesp.2022.392.10889 (PMC11397577; doi:10.17843/rpmesp.2022.392.10889)
Supplement: Translation

# Translation: Infecciones de transmisión sexual en mujeres con problemas de fertilidad en el estado Aragua, Venezuela

**Authors:** César Pacheco, Jham Lugo, Narviz Pulido, Heriberto Correia, Nancy Moreno, Flor Herrera

**Affiliations:** 1 Instituto de Investigaciones Biomédicas (BIOMED) “Dr. Francisco J. Triana Alonso”, Facultad de Ciencias de la Salud, Núcleo Aragua, Universidad de Carabobo, Venezuela.; 2 Centro de Fertilidad CenFer, Clinica Lugo, Maracay, Estado Aragua, Venezuela.

Sr. Editor. Algunas infecciones de transmisión sexual (ITS) pueden causar infertilidad en las mujeres ^1^. En Venezuela existen pocos reportes publicados sobre la proporción de estas infecciones en mujeres con problemas de fertilidad, por lo tanto, existe la necesidad de generar conocimiento sobre este tema. El propósito de este estudio fue determinar la proporción de las ITS por *C. trachomatis, T. vaginalis, N. gonorrhoeae, *y *Mycoplasmas *(*M. hominis, M.genitalium y U. urealyticum)* en un grupo de mujeres con problemas de fertilidad atendidas en el Centro de Fertilidad (CenFer) ubicado en la ciudad de Maracay, estado Aragua durante los años 2016-2019.

Se realizó un estudio transversal, con una muestra de selección continua y un muestreo no probabilístico. La infertilidad se definió, según la Organización Mundial de la Salud (OMS) ^2^. Se incluyeron a mujeres heterosexuales con actividad sexual, en edad reproductiva e infertilidad primaria o secundaria, nuligestas, con abortos previos o anembrionados o con hijos previos. Se excluyeron a mujeres en tratamiento con antibióticos siete días antes de la toma de muestra. 

La muestra de hisopado cervical fue trasladada desde el CenFer al Instituto de Investigaciones Biomédicas (BIOMED) para el diagnóstico molecular. El ADN de los hisopos se extrajo por el método fenol-cloroformo, su integridad se determinó por electroforesis en geles de agarosa al 1% y su concentración por absorbancia a 260 nm. La amplificación del ADN se realizó utilizando el método descrito por el fabricante del estuche comercial Seeplex® STD6 ACE detection (Seegene, Seoul, Korea). Este estuche contiene seis pares de cebadores específicos para detectar genes únicos de los patógenos *C. trachomatis, T. vaginalis, N. gonorrhoeae, M. hominis, M. genitalium y U. urealyticum; *se incluyen controles positivos de ADN. Los productos de amplificación se visualizaron por electroforesis en geles de agarosa al 2%. La muestra se consideró positiva si presentó un patógeno, negativa si no tuvo ninguno y mixta si presentó dos o más patógenos.

Se calcularon las distribuciones de frecuencias absolutas y relativas para los resultados de las pruebas de identificación de especies y para comparar la proporción de cada una de las infecciones entre los años estudiados, se aplicó la prueba de homogeneidad de chi cuadrado (χ^2^). Los datos se procesaron con el *software* estadístico RStudio 1.1.463.

El promedio de edad fue de 33,6 ± 7,8 años con un rango de 18 a 50 años. En la Tabla 1 se muestra los porcentajes de muestras positivas, mixtas y negativas, y la distribución de la frecuencia de los patógenos en las muestras positivas más mixtas, durante el período 2016-2019. La prueba de homogeneidad de χ^2 ^indicó que las proporciones anuales de muestras positivas y negativas, así como las especies identificadas, no fueron homogéneas para el período considerado (χ^2^=35,96; p<0,001 y χ^2^= 33,36; p=0,003, respectivamente).

**Tabla 1:** Proporción y distribución de los patógenos detectados en mujeres con problemas de fertilidad durante el período 2016-2019.

	2016 n=125 (%)	2017 n=144 (%)	2018 n=101 (%)	2019 n=123 (%)	Total N=493 (%)	Valor de p ^a^
Muestra						
Positiva	80 (64,0)	86 (59,7)	35 (34,7)	55 (44,7)	256 (51,9)	<0,001
Mixta	12 (9,6)	20 (13,9)	9 (8,9)	8 (6,5)	49 (9,9)	
Negativa	33 (26,4)	38 (26,4)	57 (56,4)	60 (48,8)	188 (38,2)	
Patógenos en las muestras positivas más mixtas						
*M. hominis*	29 (23,2)	32 (22,2)	19 (18,9)	33 (26,8)	113 (22,9)	0,034
*U. urealyticum*	32 (25,6)	39 (27,1)	10 (9,9)	5 (4,1)	86 (17,4)	0,001
*C. trachomatis*	18 (14,4)	15 (10,4)	6 (5,9)	16 (13,0)	55 (11,2)	0,392
*N. gonorrhoae*	6 (4,8)	9 (6,3)	1 (0,9)	3 (2,4)	19 (3,9)	0,676
*T. vaginalis*	4 (3,2)	7 (4,9)	3 (2,9)	5 (4,1)	19 (3,9)	0,922
*M. genitalium*	3 (2,4)	4 (2,7)	5 (4,9)	1 (0,8)	13 (2,6)	0,138

a Prueba de homogeneidad de chi cuadrado.

El porcentaje de muestras positivas fue mayor en el 2016 (64,0%) y 2017 (59,7%) y disminuyó en el 2018 (34,7%) y 2019 (44,7%). Durante el periodo 2016-2017 hubo un desabastecimiento de insumos sanitarios, y en el periodo 2018-2019 hubo una mayor importación de estos insumos ^3^. Muy posiblemente, los resultados de este trabajo estuvieron influidos por factores sociopolíticos-económicos ocurridos en Venezuela durante el periodo 2016-2019 ^3^. Sería necesario realizar estudios retrospectivos para corroborar estos hallazgos. 

El porcentaje de *C. trachomatis* fue de 11,2%, similar a lo hallado en un estudio realizado en mujeres infértiles (11,5%) ^4^, lo cual sugiere una participación de este patógeno en la infertilidad de algunas pacientes. Los porcentajes de *N. gonorrhoeae, M. genitallum*, y *T. vaginalis* fueron inferiores a lo hallado en otros estudios con mujeres infértiles. Para *U. urealyticum* y *M. hominis*, la literatura describe discrepancias. En un estudio en mujeres infértiles, el porcentaje de *U. urealyticum* y *M. hominis* fue de 51,7 y 26,7%, respectivamente ^5^. Otro estudio en mujeres infértiles y fértiles dio porcentajes bajos: *U. urealyticum* (6,6% infértil y 2,5% fértil) y *M. hominis *(5,3% infértil y 1,5% fértil) ^6^. En nuestro estudio, el porcentaje fue de 17,4% para *U. urealyticum* y 22,9% para *M. hominis*, estos valores se asemejan más al grupo de mujeres infértiles. Además, estos dos últimos patógenos fueron los únicos que tuvieron una variación anual estadísticamente significativa, *U. urealyticum* disminuyó con el tiempo mientras que *M. hominis* aumentó. Sería importante determinar la causa de este comportamiento.

Como limitación se debe reconocer que la muestra estaba constituida solo por mujeres con problemas de fertilidad, por lo cual los datos no pueden generalizarse a toda la población de mujeres venezolanas.

Finalmente, las muestras positivas para ITS fueron mayores en los dos primeros años del período 2016-2019. Esto podría tener múltiples causas como una fuerte escasez de medicamentos debido a la crisis sociopolítico-económica del país ^3^, el aumento en el número de parejas sexuales o una causa tan simple como falta de interés en la consulta uroginecológica oportuna y preventiva. De todas estas posibilidades, solo se puede afirmar que durante los años 2016-2017 hubo un 80% de desabastecimiento en los insumos de salud ^3^ y esto podría haber influido en un aumento de las ITS.
